# Multidimensional Deep Learning Reduces False-Positives in the Automated Detection of Cerebral Aneurysms on Time-Of-Flight Magnetic Resonance Angiography: A Multi-Center Study

**DOI:** 10.3389/fneur.2021.742126

**Published:** 2022-01-18

**Authors:** Yuki Terasaki, Hajime Yokota, Kohei Tashiro, Takuma Maejima, Takashi Takeuchi, Ryuna Kurosawa, Shoma Yamauchi, Akiyo Takada, Hiroki Mukai, Kenji Ohira, Joji Ota, Takuro Horikoshi, Yasukuni Mori, Takashi Uno, Hiroki Suyari

**Affiliations:** ^1^Graduate School of Science and Engineering, Chiba University, Chiba, Japan; ^2^Department of EC Platform, ZOZO Technologies, Inc., Tokyo, Japan; ^3^Department of Diagnostic Radiology and Radiation Oncology, Graduate School of Medicine, Chiba University, Chiba, Japan; ^4^Department of Radiology, Chiba University Hospital, Chiba, Japan; ^5^Graduate School of Engineering, Chiba University, Chiba, Japan

**Keywords:** deep learning, cerebral aneurysm, magnetic resonance angiography, false positive, autodetection, multidimensional convolutional neural network

## Abstract

Current deep learning-based cerebral aneurysm detection demonstrates high sensitivity, but produces numerous false-positives (FPs), which hampers clinical application of automated detection systems for time-of-flight magnetic resonance angiography. To reduce FPs while maintaining high sensitivity, we developed a multidimensional convolutional neural network (MD-CNN) designed to unite planar and stereoscopic information about aneurysms. This retrospective study enrolled time-of-flight magnetic resonance angiography images of cerebral aneurysms from three institutions from June 2006 to April 2019. In the internal test, 80% of the entire data set was used for model training and 20% for the test, while for the external tests, data from different pairs of the three institutions were used for training and the remaining one for testing. Images containing aneurysms > 15 mm and images without aneurysms were excluded. Three deep learning models [planar information-only (2D-CNN), stereoscopic information-only (3D-CNN), and multidimensional information (MD-CNN)] were trained to classify whether the voxels contained aneurysms, and they were evaluated on each test. The performance of each model was assessed using free-response operating characteristic curves. In total, 732 aneurysms (5.9 ± 2.5 mm) of 559 cases (327, 120, and 112 from institutes A, B, and C; 469 and 263 for 1.5T and 3.0T MRI) were included in this study. In the internal test, the highest sensitivities were 80.4, 87.4, and 82.5%, and the FPs were 6.1, 7.1, and 5.0 FPs/case at a fixed sensitivity of 80% for the 2D-CNN, 3D-CNN, and MD-CNN, respectively. In the external test, the highest sensitivities were 82.1, 86.5, and 89.1%, and 5.9, 7.4, and 4.2 FPs/cases for them, respectively. MD-CNN was a new approach to maintain sensitivity and reduce the FPs simultaneously.

## Introduction

Cerebral aneurysms are found in 2–3% of the population and are a major cause of subarachnoid hemorrhage (SAH), with high mortality and morbidity (1–3). Early detection of unruptured cerebral aneurysms can prevent SAH. Time-of-flight magnetic resonance angiography (TOF-MRA) is often used as a screening tool for the detection of unruptured aneurysms and is frequently used in patients with other cerebral issues. The sensitivity of TOF-MRA was found to be 95% in a meta-analysis (4). Conventional screening for cerebral aneurysms requires considerable time and effort, even for experienced radiologists, and several studies have reported differences in the sensitivity of aneurysm detection depending on various conditions (e.g., the location and size of the aneurysm, and the radiologist's years of experience and proficiency) (5–7).

Various studies have been conducted on how to assist radiologists in their routine interpretation of images by automatically detecting aneurysms using machine learning (8–10). Recently, convolutional neural networks (CNNs) using TOF-MRA images as input have been implemented widely (7, 11–16). Several studies have demonstrated that CNN-based aneurysm detection techniques based on planar information achieved higher sensitivity and versatility in external datasets than conventional machine learning approaches (11, 12). A CNN-based computer-aided diagnostic (CAD) system for aneurysm detection achieved high sensitivity (91–93%); however, the system yielded too many false-positives (FPs; 5–9 per case), with few true positives (12). Systems yielding high FPs can make CAD unreliable and can increase the radiologist's interpretation burden. However, approaches for improving CNN to reduce the FPs have not been fully explored.

We hypothesized that the numerous FPs in CNN-based systems could be produced because previous studies did not fully exploit the stereoscopic structure of arteries in TOF-MRA and did not provide their systems with sufficient clues to distinguish between positive and negative cases. In fact, the bifurcation or curvature of the artery was misidentified as an aneurysm in some previous studies (11, 14).

Therefore, we developed a new technique for effective utilization of the planar and stereoscopic structure of TOF-MRA in a CAD system with a CNN, to reduce the FPs. Our model is a simple extension of the popular CNN using only planar input, and it can be easily applied to various architectures to reduce FPs. We evaluated our model on TOF-MRA images acquired from various medical institutions and with different imaging device conditions, to validate its versatility and reproducibility.

## Materials and Methods

Our institutional review board approved this retrospective study and waived the requirement to obtain written informed consent. The information for this study was displayed on our institutional home page and in the waiting room of our department. All participants were given the opportunity to opt-out of this study.

### Data Sets

**Figure 1:**
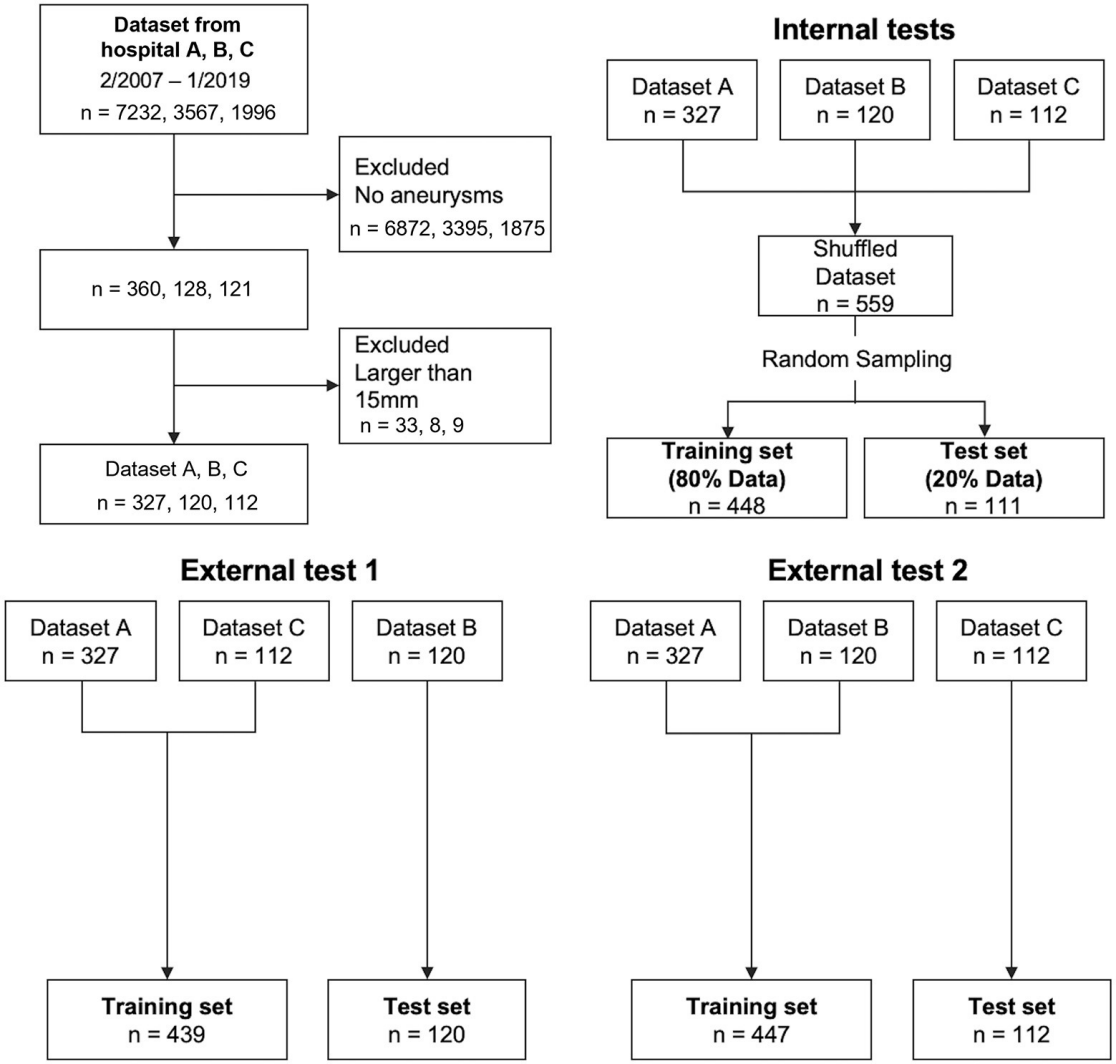
Flowcharts of subject selection and test patterns.

TOF-MRA images of newly diagnosed aneurysms, acquired between June 2006 and April 2019 at three hospitals, were included. Aneurysm diagnosis was initially performed by each institution's board-certified radiologists and was confirmed by another board-certified radiologist (^**^ with 15 year's experience in neuroradiology) for this study. Three board-certified radiologists (^**^, ^**^, and ^**^ with 4-, 4- and 15-year's experience in neuroradiology) delineated the volumes-of-interest of aneurysms on TOF-MRA images, in consensus. Images containing aneurysms > 15 mm, which were unlikely to be overlooked by radiologists during interpretation, were excluded. Images were divided into datasets for internal and external tests (Figure 1). The location of the aneurysm was classified. Aneurysm at the junction of the internal carotid and posterior communicating arteries was included in the internal carotid artery category. The category of the posterior communicating artery was the aneurysm far from the internal carotid artery.

### Image Processing Methods

#### Artery Extraction

Data were normalized to reduce bias. TOF-MRA images were resampled to produce isotropic slices (0.3906 mm), and the signal values were normalized by the Nyúl and Udupa normalization method (17). Threshold processing was applied to remove non-arterial tissues from the images. The three-sigma signal level was empirically used as the threshold value.

#### Candidate Voxel Extraction

Curvature and bifurcation, candidate features for aneurysm detection, were extracted using the selective enhancement filter (18), which is composed of blob-like, line-like, and plane-like shape-enhancement filters. The blob-like shape-enhancement filter was leveraged to enhance the blob-like shape from the artery image, and the enhanced points were considered as candidates of aneurysm. Finally, the voxels were cropped around the points that were extracted in the previous step and were labeled as positive if the voxel contained an entire aneurysm. Voxels containing a part of the aneurysm were discarded during model training. Then, some voxels that did not include any aneurysms were labeled as negative and excluded using the gradient-boosting decision-tree classifier (19). The classifier was trained on some image features, such as curvature or sphericity computed manually from the TOF-MRA image. With the above method, many small voxels of lesion candidate are extracted from a patient. Thus, the number of training data becomes larger and the model can train with a limited number of patients.

### Maximum Projection Method (MIP) Image Generation

To develop a model with a planar input, the MIP was applied to voxels along 15 axes, and the 15 MIP images were then simply concatenated vertically for planar input models. Each image was obtained by applying MIP, and after rotating the voxels by 30 and 45° on the X, Y, and Z axes. Supplementary Figure 1 in the supplementary materials shows the procedures for acquiring MIP images from a single voxel.

### Model Development

**Figure 2:**
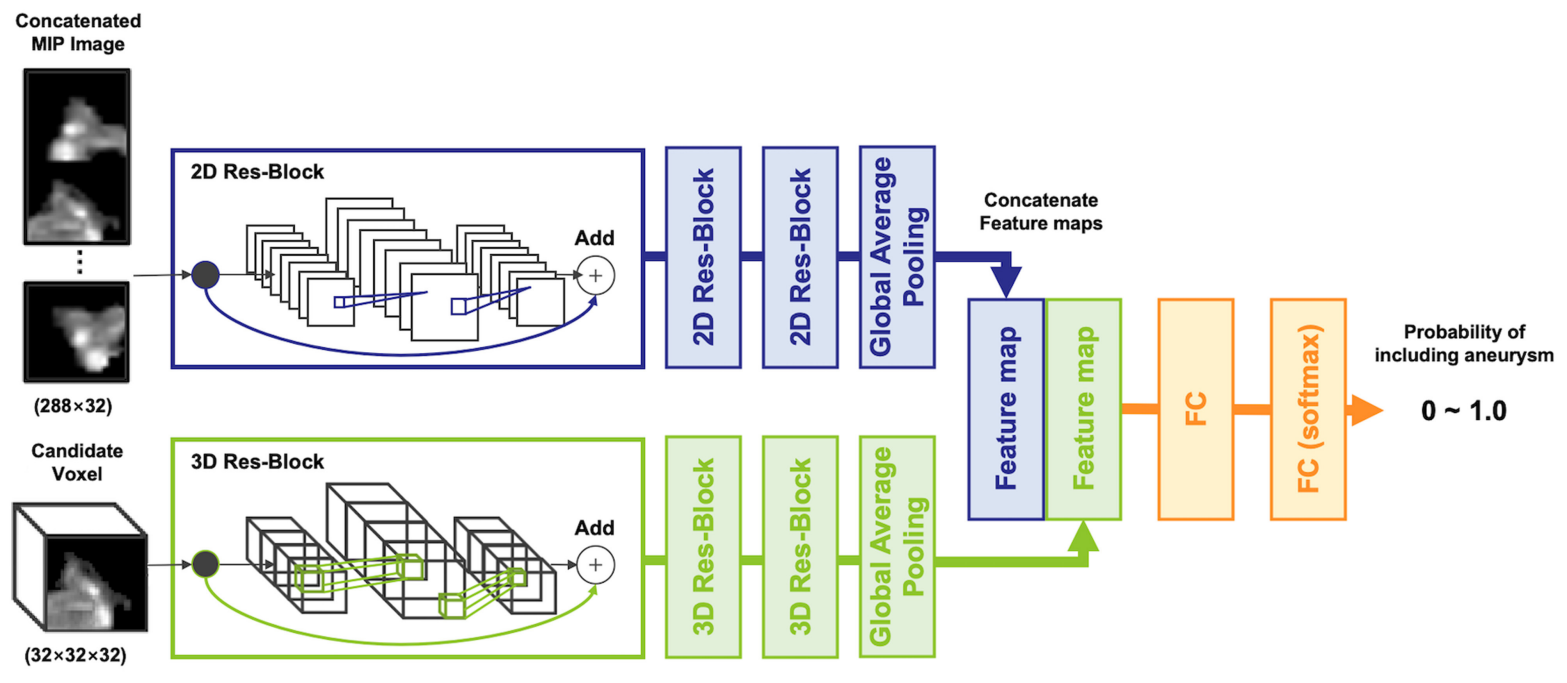
The architecture of the multidimensional convolutional neural network, including 2D and 3D-CNN. A concatenated maximum intensity projection image as input into the 2D-CNN was generated from a candidate voxel.

To input multiple spatial features of arteries and aneurysms into the model, a two-input network, multidimensional CNN (MD-CNN), which processes two-dimensional plane and three-dimensional stereoscopic features, was developed (Figure 2), and consisted of a combination of planar (2D-CNN) and stereoscopic CNN (3D-CNN). The 2D-CNN handled an MIP image generated from TOF-MRA, and 3D-CNN handled raw voxels extracted from the TOF-MRA image. The outputs of both the 2D-CNN and 3D-CNN were compressed and coupled by a global average pooling layer, to utilize features from both networks, and were finally connected to a fully connected layer, to output the probability of an aneurysm being present. Each CNN was based on SE-ResNet (20, 21) and trained from scratch, simultaneously. Four-fold cross-validation was used to determine network hyperparameters, such as layer depth and learning rate, of each CNN with a training dataset. Model development hyperparameters are described in the Supplementary Materials. When detecting aneurysms, the candidate voxel extracted in the previous phase was fed into the model, and the probability of being positive inside the voxel was obtained. If the probability was >0.5, the region surrounded by the voxel was considered as the positive candidate region. The part of the artery determined as a positive candidate region by more than five candidate voxels was then considered positive; otherwise, it was considered negative. The number of voxels considered as positive was determined empirically.

### Model Evaluation

In the internal test, 20% of all patients were randomly selected for test data, and the remaining 80% were used as training data. Of the latter, 20% were used as validation data to track the learning progress (Figure 1). In the external test, we examined models based on external data that were not included in the training or validation data, to investigate general model versatility, in two experiments, using different data. In external test 1, data from hospitals A and C were used as training data, and data from hospital B were used as testing data. In external test 2, data from hospitals A and B were used as training data, and data from hospital C as testing data. Validation data in both external tests were randomly selected from the training data, similar to the internal test.

### Data Set Characteristics

The data set characteristics are summarized in Table 1, Supplementary Table 1. In the internal test, 448 cases were used for training and 111 for testing. In external test 1, 439 cases were used for training and 120 for testing. In external experiment 2, 447 cases were used for training and 112 cases for testing.

**Table 1:** Data set characteristics of the internal test and two external tests.

**Characteristic**	**Internal Test**		**External Test 1**		**External Test 2**	
	**Training set**	**Test set**	**Training set**	**Test set**	**Training set**	**Test set**
Examinations	448	111	439	120	447	112
Male	163 (36.4)	42 (37.8)	165 (37.6)	40 (33.3)	172 (38.5)	33 (29.5)
Female	285 (63.6)	69 (62.2)	274 (62.4)	80 (66.7)	275 (61.5)	79 (70.5)
Age (y)^*^	71 ± 12	71 ± 12	71 ± 12	72 ± 11	70 ± 11	76 ± 12
Male	70 ± 10	72 ± 8	70 ± 10	71 ± 11	70 ± 9	73 ± 12
Female	72 ± 12	70 ± 14	71 ± 13	72 ± 10	70 ± 13	77 ± 12
Aneurysms	589	143	576	156	570	162
Mean size of aneurysms (mm)^*^	5.9 ± 2.3	6.2 ± 2.9	5.9 ± 2.5	5.9 ± 2.3	5.8 ± 2.4	6.2 ± 2.6
**Locations**
Internal carotid artery	285 (48.4)	66 (46.2)	264 (45.8)	87 (55.8)	305 (53.5)	46 (28.4)
Middle cerebral artery	161 (27.3)	40 (28.0)	167 (29.0)	34 (21.8)	117 (20.5)	84 (51.9)
Anterior cerebral artery	42 (7.1)	11 (7.7)	50 (8.7)	3 (1.3)	43 (7.5)	10 (6.2)
Anterior communicating artery	47 (8.0)	13 (9.1)	44 (7.6)	16 (10.3)	45 (7.9)	15 (9.3)
Basilar artery	35 (5.9)	8 (5.6)	32 (5.6)	11 (7.1)	38 (6.7)	5 (3.1)
Vertebral artery	7 (1.2)	2 (1.4)	7 (1.2)	2 (1.3)	8 (1.4)	1 (0.6)
Posterior cerebral artery	8 (1.4)	2 (1.4)	8 (1.4)	2 (1.9)	9 (1.6)	1 (0.6)
Superior cerebellar artery	4 (0.7)	1 (0.7)	4 (0.7)	1 (0.6)	5 (0.9)	0
**Magnetic field strength**
1.5-T	382 (64.9)	87 (60.8)	449 (78.0)	20 (12.8)	307 (53.9)	162 (100)
3.0-T	207 (35.1)	56 (39.2)	127 (22.0)	136 (87.2)	263 (46.1)	0

*Data in parentheses are percentages*.

* *Data are mean ± standard deviation*.

### Statistical Analysis

Models were evaluated based on free-response operating characteristic (FROC) curve analysis of the number of FPs per case (No. of FPs/case) and detection sensitivity in the internal and external test data sets. The number of aneurysms detected by a model was computed separately in terms of location, size, magnetic field strength, and TOF-MRA imaging platform. The number of missing aneurysms was similarly computed. Sensitivity represents the number of true positives over the total number of aneurysms, and the No. of FPs/case represents the ratio of the number of FPs case tested to the total number of cases. The area determined to be positive was counted as true positive if it contained a part of the aneurysm, and as FP if it did not. If lesion candidates determined to be positive by the model were 16 voxels (about 6.25 mm) from each other, they were counted as different points. To evaluate the performance of the MD-CNN in terms of reducing FPs, CNNs with only two-dimensional input and only three-dimensional input were also trained and tested as the performance baseline of conventional CNNs. Models were compared in terms of the No. of FPs/case at the highest sensitivity, the No. of FPs/case at a fixed sensitivity (80%), and the sensitivity at a fixed No. of FPs/case of 3 FPs/case. In the internal test, all models were evaluated using randomly sampled test data. In the external test, all models were evaluated in two experiments, based on two unique institutions. All statistical analyses were performed using Python (version 3.6.7, https://www.python.org) and its open-source library, scikit-learn (version 0.19.1, https://scikit-learn.org).

## Results

### Characteristics of Patients and Aneurysms

Overall, 360 of 7,232 examinations in hospital A, 128 of 3,567 examinations in hospital B, and 121 of 1996 examinations in hospital C were initially diagnosed as including aneurysms (Figure 1). After excluding examinations involving aneurysms larger than 15 mm, 559 examinations containing at least one cerebral aneurysm were included.

### Model Performances

**Figure 3:**
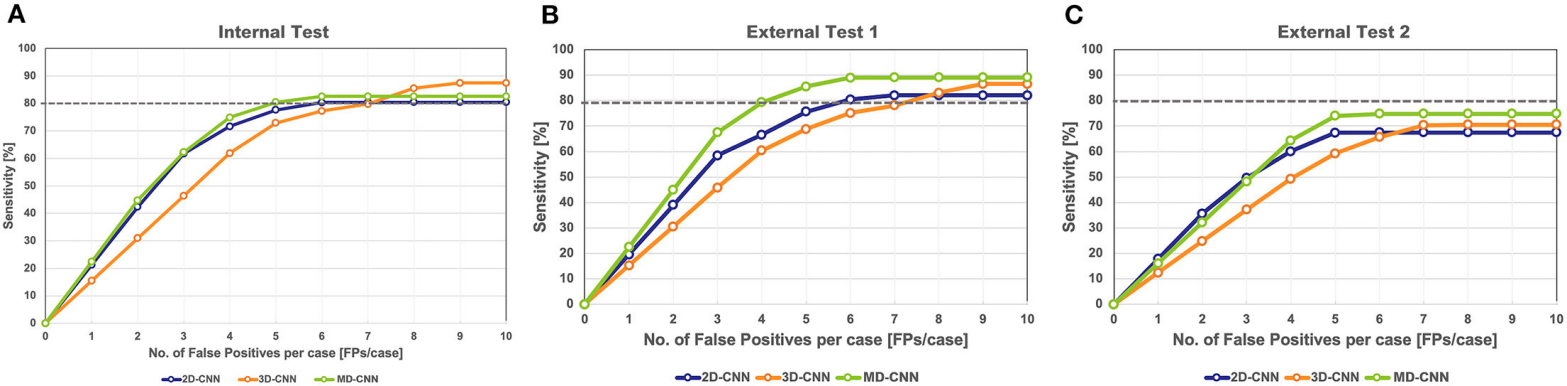
Free-response receiver operating characteristic curve of all models on the internal test **(A)** and the external test 1 **(B)** and 2 **(C)**. The gray dashed line in the graph indicates a sensitivity of 80%. CNN, convolutional neural network, MD, multidimensional.

The FROC curves for the internal test of each model are shown in Figure 3A. In the internal test, the highest sensitivities of the 2D-CNN, 3D-CNN, and MD-CNN were 80.4% (115 of 143) at 6.1 FPs/case, 87.4% (125 of 143) at 8.5 FPs/case, and 82.5% (118 of 143) at 5.4 FPs/case, respectively. Although the 3D-CNN achieved the highest sensitivity, the MD-CNN achieved the least FPs at a sensitivity of 80%, with 5.0 FPs/case, compared to the 2D-CNN and 3D-CNN with 6.1 and 7.1 FPs/case, respectively.

In external test 1, the highest sensitivities of 2D-CNN, 3D-CNN, and MD-CNN were 82.1% (128 of 156) at 6.6 FPs/case, 86.5% (135 of 156) at 8.8 FPs/case, and 89.1% (139 of 156) at 6.1 FPs/case, respectively. All models yielded higher sensitivities than obtained in the internal test; in particular, the sensitivity of the MD-CNN increased significantly by 6% over that in the internal test, with only a 0.7 FPs/case increase. The FPs at 80% sensitivity was 5.9 FPs/case for the 2D-CNN, 7.4 FPs/case for the 3D-CNN, and 4.2 FPs/case for the MD-CNN. In external test 1, the MD-CNN yielded the highest sensitivity and also showed the least FPs at 80% sensitivity (Figure 3B).

In external test 2, the highest sensitivities of the 2D-CNN, 3D-CNN, and MD-CNN were 67.3% (109 of 162) at 5.0 FPs/case, 70.4% (114 of 162) at 7.1 FPs/case, and 74.7% (121 of 162) at 5.3 FPs/case, respectively. None of the models had a sensitivity of <80%; however, the MD-CNN had a higher sensitivity, with a fewer FPs than the other two models (Figure 3C).

**Figure 4:**
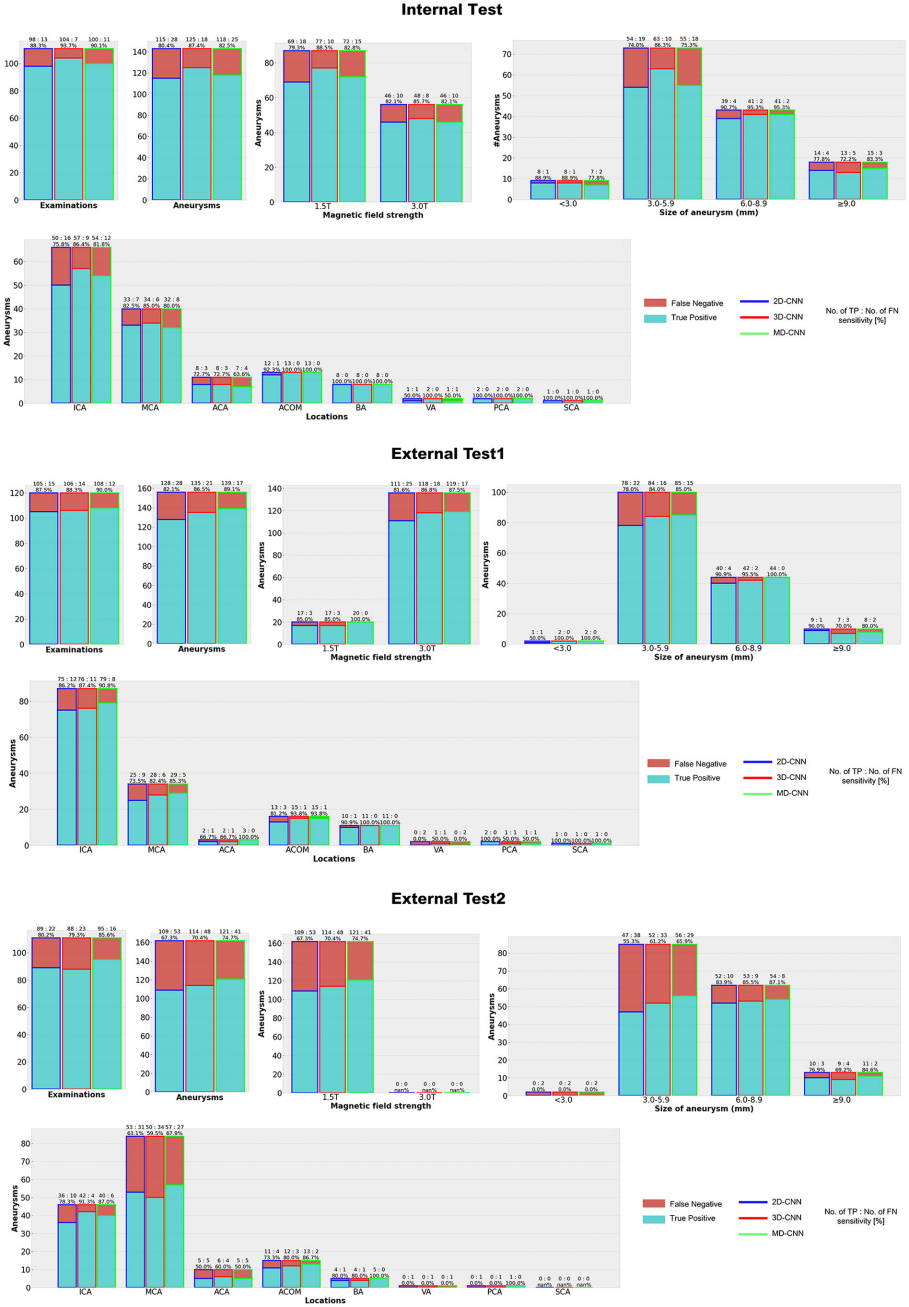
Detection results with each deep learning model. CNN, convolutional neural network, MD, multidimensional, ICA, Internal carotid artery, MCA, Middle cerebral artery, ACA, Anterior cerebral artery, ACOM, Anterior communicating artery, BA, Basilar artery, VA, Vertebral artery, PC, Posterior cerebral artery, SCA = Superior cerebellar artery.

Figure 4, Supplementary Table 2 show the detection results of the internal and external tests. All models showed similar trends in terms of the size of the aneurysms, with the highest sensitivity for 6.0–8.9-mm aneurysms throughout all tests, while relatively small aneurysms (3.0–5.9-mm) tended to be missed, particularly in external test 2 with a sensitivity of 55–66%. In terms of aneurysm location, all models detected aneurysms in the anterior communicating artery and basilar artery with high sensitivity (>80%), except for 2D-CNN in external test 2, and with a comparatively low sensitivity for those in the anterior cerebral artery.

**Figure 5:**
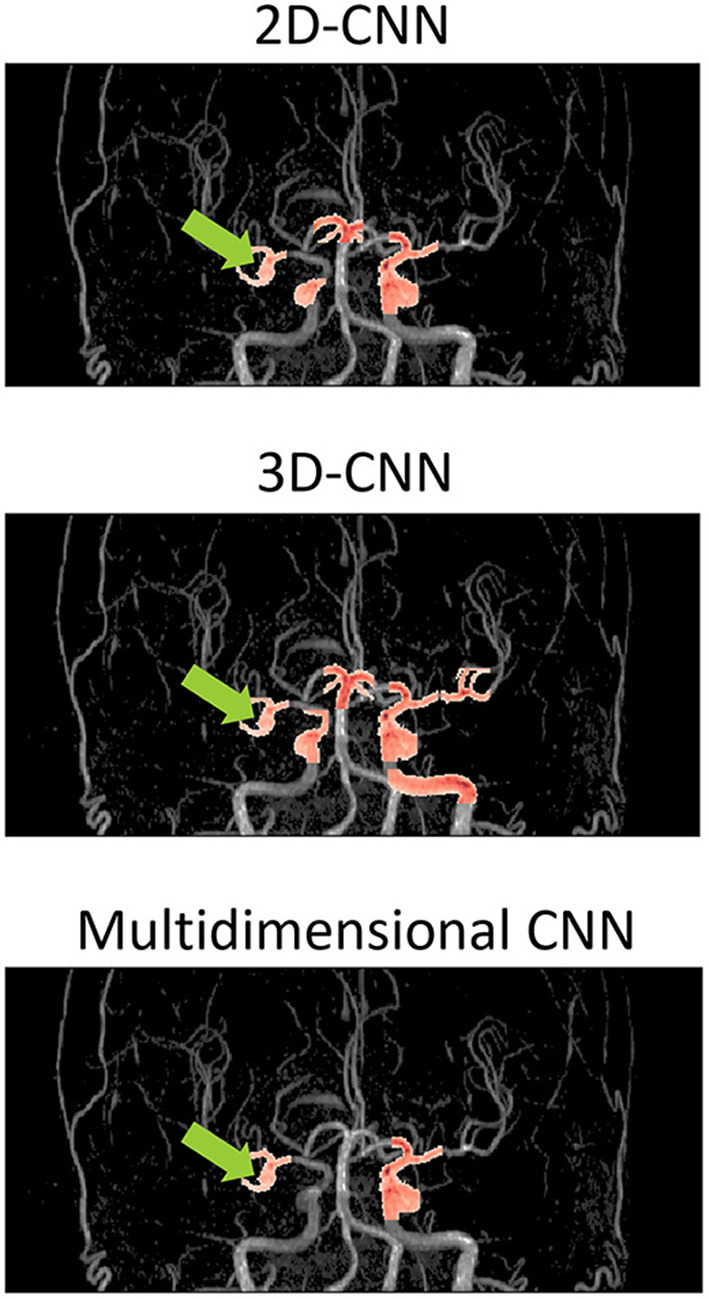
Examples of detection results of each model overlaid on the magnetic resonance angiography images. The images are from the coronal view. The tip of the light green arrow represents the aneurysm, and the red area represents where each model recognized positive candidates. The red area includes both false positives and true positives. The red area indicated by the light green arrow is a true positive, and the others red areas are false positives. The 2D-model and 3D-model represented 5 and 9 FPs, respectively, while MD-model output represented only 3 FPs. CNN, convolutional neural network.

**Figure 6:**
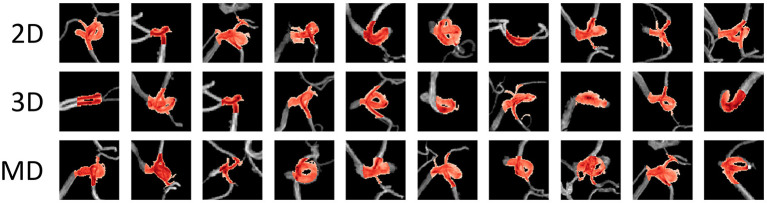
Examples of false-positive areas cut out from magnetic resonance angiography images. The red area represents the area detected as positive. These false-positives were randomly sampled from each detection result and there is no correspondence among columns. MD, multidimensional.

Figure 5 shows examples of the aneurysm detection results in each model. All models detected the right middle cerebral artery as a positive candidate. However, the MD-model had fewer FPs than the 2D-model and 3D-model. Some examples of FPs detected by each model are shown in Figure 6. FPs are often seen at the bifurcation or refraction of the blood vessels.

## Discussion

### Summary of Findings

We developed a deep learning-based model for reducing the FPs in automated cerebral aneurysm detection, and tested it under various conditions to verify the sensitivity and FPs. Our MD-CNN, which handles planar and stereoscopic information, had a fewer FPs and higher sensitivity than the simple 2D-CNN and 3D-CNN. In the internal test, at 80% sensitivity, the MD-CNN reduced the FPs by 1.1 and 2.1 FPs/case when compared with the 2D-CNN and 3D-CNN, respectively. In external test 1, the MD-CNN also produced 1.7 and 3.2 fewer FPs/case than the 2D-CNN and 3D-CNN, respectively, at 80% sensitivity. Although all models decreased sensitivity in external test 2, the MD-CNN yielded the highest sensitivity, with a fewer FPs than the other models. Thus, our simple extension of the deep learning model can easily reduce the FP detection of cerebral aneurysms.

The sensitivity of the MD-CNN for 6.0–8.9-mm aneurysms in both internal and external test 1 exceeded 90%; however, its sensitivity was about 10% lower in external test 2, and its sensitivity for 3.0–5.9-mm aneurysms was 10–20% lower, which may be due to image quality. In external test 2, TOF-MRA images acquired with only the 1.5-T imaging platforms were used, which may have caused poor model performance when compared with the 3-T image data. The sensitivity of external test 1, mostly involving 3-T data, was higher than that of the internal test, suggesting that the magnetic field strength affected the sensitivity and FPs.

Each model had a different tendency in terms of the shape of arteries that were incorrectly identified (Figure 6). All models tended to recognize voxels that include many narrow arteries as positive, and in some cases, the 2D-CNN recognized voxels with only large arteries, such as the internal carotid artery (ICA) flexure, as positive. This may be because the 2D-CNN used MIP images acquired from various viewpoints, and the angle may have caused features to appear as an aneurysm. The MD-CNN suppressed such FPs; however, it often mistakenly detected areas diverging from the ICA, such as the posterior communicating artery bifurcation. Thus, providing the model with three-dimensional information may enhance its sensitivity to features that could be misinterpreted.

Generally, it is difficult to optimize 3D-CNNs without overfitting, due to the large number of parameters, and require validation under various data conditions. In the internal test, our 3D-CNN detected 87.4% of aneurysms, with 8.5 FPs; however, sensitivity was significantly reduced (by 17%) in external test 2. This suggests that training the model with only three-dimensional information remains challenging in medical applications. Consequently, we employed features derived from 3D-CNN as auxiliary features, using them for training simultaneously with 2D-CNN features. In this way, our MD-CNN could be trained without overfitting, and could correctly identify areas falsely appearing to be aneurysms (slight protrusions or curvatures of the arteries) asnon-aneurysms.

### Comparison With Existing Knowledge

The automatic detection of cerebral aneurysms has been attempted by various modalities, including computed tomography (CT) angiography, digital subtraction angiography, and MRA. However, CT angiography and digital subtraction angiography are often performed in patients with suspected or confirmed cerebral aneurysms. On the other hand, MRA is a sequence that is often included in routine brain MRI examinations, and cerebral aneurysms are frequently detected incidentally. Therefore, the risk of oversight is high. Nakao et al. (11) developed a 2D-CNN-based model that takes multiple images from various directions created by the maximum intensity projection. Ueda et al. (12) developed a 3D-CNN-based model that takes three-dimensional patches from aneurysm candidates. Their approaches respectively resemble 2D-CNN and 3D-CNN in this study.

Nakao et al. developed a 2D-CNN-based model with a sensitivity of 94.2% and FPs of 2.9 FPs/case, which is better than our method (11). However, because of the different data sets and evaluation criteria, a simple comparison is impossible. Moreover, in the Nakao's method, training and testing were conducted using only the data of a single hospital, and external testing was not conducted. Our method has an important advantage over their work in the actual applications and usefulness using data from multiple institutions and with external testing.

Three-dimensional structural information has increasingly been used in CAD for aneurysm detection. Joo et al. (22) developed a 3D-CNN-based model to detect aneurysms using TOF-MRA images, with high sensitivity and specificity in 106 external datasets, including examinations without aneurysms. Faron et al. (23) reported that the combination of radiologists and 3D-CNN-based model improved the detection rate of aneurysms.

To prevent oversight, a certain number of FPs are acceptable as long as high sensitivity is achieved, but FPs directly lead to prolonged reading time. Reliable lesion prediction with few FPs is essential for creating a CAD system that supports routine interpretation in daily practice. FP reduction techniques have been widely reported in lung nodule-detection tasks (24–32). For cerebral aneurysm detection, some studies have shown that the gradient-boosting algorithm or rule-based schemes can remove FPs with heuristic shape-based features (33, 34); however, no efforts to extend the CNN structure for FP reduction by improving the structure of the deep learning model have been reported in this field to date.

### Strengths and Limitations

The strength of this study is that our strategy using MD-CNN was a novel approach to detect cerebral aneurysm and to reduce FPs. Moreover, the collected data included four 3.0 T and ten 1.5 T MRIs of three vendors from three institutes. Confounders related to MRI, such as magnitude strength and manufacturer, can degrade the generalization performance of the deep learning model. When using deep learning models in clinical practice, there is a need for a deep learning model that can withstand various environments. Our model is one of them. Still, our study had some limitations. First, all data used for training and testing contained at least one aneurysm; we also need to verify the FPs for images lacking aneurysms. This theme is likely to be the next topic of automated detection of cerebral aneurysm. Second, the datasets used for training and external testing lacked diversity, particularly in terms of aneurysm size. Since we did not have a sufficient number of small aneurysms for model training, more such cases could further improve the sensitivity and FPs for small aneurysms. Third, the exclusion of aneurysms >15 mm may have decreased the generalizability of our method. Fourth, to improve model parameter optimization, various methods of three-dimensional convolution have been reported in the field of three-dimensional object recognition to date, and could be used to optimize our model parameters more efficiently. Finally, further studies are needed to evaluate the clinical utility of this system in comparison to, or combination with, humans.

In conclusion, our MD-CNN, which combines a 2D-CNN and 3D-CNN, reduced the FPs in aneurysm detection in TOF-MRA images in internal and external tests than the conventional two- and three-dimensional models, while maintaining a high sensitivity. Eliminating FPs while maintaining high sensitivity will reduce the number of candidate positives to be confirmed in routine diagnosis in daily practice, improve the reliability of CAD, and further reduce the burden of interpretation for radiologists.

## Data Availability Statement

The raw data supporting the conclusions of this article will be made available by the authors, without undue reservation.

## Ethics Statement

The studies involving human participants were reviewed and approved by Institutional Review Board of Chiba University Graduate School of Medicine. Written informed consent for participation was not required for this study in accordance with the national legislation and the institutional requirements.

## Author Contributions

YT and HY designed the study. YT, HY, and KT analyzed and interpreted the data. TM, TT, and RK annotated the data. SY, AT, HM, and KO collected the MRI data. YT, HY, KT, JO, and TH drafted the manuscript. YM, TU, and HS supervised this study. All authors contributed to the article and approved the submitted version.

## Funding

This study is funded by Inohana Shogakukai Grand (Heisei 30). The sponsors played no role in the design and management of the study, collection and analysis of data, interpretation of the results or the writing of the manuscript.

## Conflict of Interest

YT is employed by ZOZO Technologies, Inc. The remaining authors declare that the research was conducted in the absence of any commercial or financial relationships that could be construed as a potential conflict of interest.

## Publisher's Note

All claims expressed in this article are solely those of the authors and do not necessarily represent those of their affiliated organizations, or those of the publisher, the editors and the reviewers. Any product that may be evaluated in this article, or claim that may be made by its manufacturer, is not guaranteed or endorsed by the publisher.
