# Supplementary material for: Multidimensional Deep Learning Reduces False-Positives in the Automated Detection of Cerebral Aneurysms on Time-Of-Flight Magnetic Resonance Angiography: A Multi-Center Study
Source: Front Neurol. 2022 Jan 18;12:742126. doi: 10.3389/fneur.2021.742126 (PMC8805516; doi:10.3389/fneur.2021.742126)
Supplement: Supplementary file 1 [file Table_1.DOCX]

**Supplemental Materials**

***Maximum projection method (MIP) Image Generation***

**
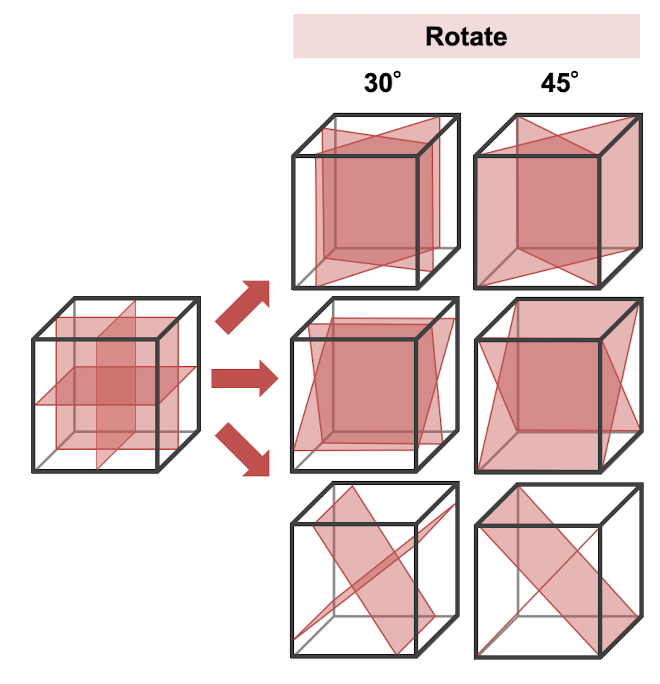
**

**Figure S1:** Fifteen maximum intensity projection (MIP) images were obtained from a single voxel. Large size MIP images, such as those with diagonal faces, were resized to make all MIP images uniform in size.

***Hyperparameters for Model Development***

The following hyperparameters were used for training: 200 epochs; base learning rate for the scratch model, Adam optimizer (lr=0.0001) (1), and decreased to 1/10 at 50% of the total epochs and to 1/10 at 75% of the total epochs; L2 weight decay of 0.0001 to the loss for all trainable parameters for layer regularization. To control for class imbalance, the negative voxels were under sampled to balance them between positive and negative voxels during the training step, and no under sampling was performed during the validation and testing steps. Our model was built on the Tensorflow framework and learned on NVIDIA GeForce GTX 1080 Ti, Intel (R) Core (TM) i3 -8350 K CPU @ 4.00 GHz and 32 GB of RAM.

**Reference**

1. Kingma DP, Ba J. Adam. A Method for Stochastic Optimization. arXiv [cs.LG]. 2014. http://arxiv.org/abs/1412.6980.

**Table S1: The demographics of the imaging platform used in each experiment.**

| **Characteristics** | | | **Internal test** | | **External test 1** | | **External test 2** | |
| --- | --- | --- | --- | --- | --- | --- | --- | --- |
| **Manufacturer / Model** | | **Magnetic field strength** | **Training set** | **Test set** | **Training set** | **Test set** | **Training set** | **Test set** |
| GE Medical Systems | |  |  |  |  |  |  |  |
|  | Signa HDxt | 3.0 T | 143 | 30 | 178 | 0 | 178 | 0 |
|  | Discovery MR750 | 1.5 T | 73 | 18 | 90 | 0 | 90 | 0 |
|  | Genesis Signa | 1.5 T | 17 | 9 | 22 | 0 | 22 | 0 |
|  | Signa EXCITE | 1.5 T | 4 | 1 | 4 | 0 | 4 | 0 |
|  | Signa Explorer | 1.5 T | 0 | 0 | 1 | 0 | 1 | 0 |
|  | Signa Creator | 1.5 T | 1 | 1 | 1 | 0 | 1 | 0 |
| Philips Medical Systems | |  |  |  |  |  |  |  |
|  | Achieva dStream | 1.5 T | 33 | 8 | 40 | 0 | 40 | 0 |
|  | Achieva | 1.5 T | 34 | 4 | 41 | 0 | 41 | 0 |
|  | Achieva Preludio | 3.0 T | 0 | 0 | 1 | 0 | 1 | 0 |
|  | Ingenia | 3.0 T | 154 | 37 | 35 | 136 | 171 | 0 |
|  | Intera | 1.5 T | 20 | 3 | 0 | 20 | 20 | 0 |
| Siemens | |  |  |  |  |  |  |  |
|  | Symphony | 1.5 T | 105 | 27 | 137 | 0 | 0 | 137 |
|  | Aera | 1.5 T | 17 | 4 | 25 | 0 | 0 | 25 |
|  | Skyra | 3.0 T | 1 | 1 | 1 | 0 | 1 | 0 |

**Table S2: Detection sensitivity of each experiment for each imaging device.**

| **Characteristics** | | | **Sensitivity [%]** | | | | | | | | |
| --- | --- | --- | --- | --- | --- | --- | --- | --- | --- | --- | --- |
|  |  |  | **Internal test** | | | **External test 1** | | | **External test 2** | | |
| **Manufacturer / Model** | | **Magnetic field strength** | **2D-CNN** | **3D-CNN** | **MD-CNN** | **2D-CNN** | **3D-CNN** | **MD-CNN** | **2D-CNN** | **3D-CNN** | **MD-CNN** |
| GE Medical Systems | |  |  |  |  |  |  |  |  |  |  |
|  | Signa HDxt | 3.0 T | 86.7 (26/30) | 93.3 (28/30) | 86.7 (26/30) | 0 | 0 | 0 | 0 | 0 | 0 |
|  | Discovery MR750 | 1.5 T | 72.2 (13/18) | 72.2 (13/18) | 77.8 (14/18) | 0 | 0 | 0 | 0 | 0 | 0 |
|  | Genesis Signa | 1.5 T | 77.8 (7/9) | 77.8 (7/9) | 77.8 (7/9) | 0 | 0 | 0 | 0 | 0 | 0 |
|  | Signa EXCITE | 1.5 T | 100.0 (1/1) | 0 (0/1) | 100.0 (1/1) | 0 | 0 | 0 | 0 | 0 | 0 |
|  | Signa Explorer | 1.5 T | 0 | 0 | 0 | 0 | 0 | 0 | 0 | 0 | 0 |
|  | Signa Creator | 1.5 T | 100.0 (1/1) | 100.0 (1/1) | 100.0 (1/1) | 0 | 0 | 0 | 0 | 0 | 0 |
| Philips Medical Systems | |  |  |  |  |  |  |  |  |  |  |
|  | Achieva dStream | 1.5 T | 100.0 (8/8) | 87.5 (7/8) | 75.0 (6/8) | 0 | 0 | 0 | 0 | 0 | 0 |
|  | Achieva | 1.5 T | 75.0 (3/4) | 100.0 (4/4) | 75.0 (3/4) | 0 | 0 | 0 | 0 | 0 | 0 |
|  | Achieva Preludio | 3.0 T | 0 | 0 | 0 | 0 | 0 | 0 | 0 | 0 | 0 |
|  | Ingenia | 3.0 T | 83.8 (31/37) | 91.9 (34/37) | 83.8 (31/37) | 81.6 (111/136) | 86.8 (118/136) | 87.5 (119/136) | 0 | 0 | 0 |
|  | Intera | 1.5 T | 100.0 (3/3) | 100.0 (3/3) | 100.0 (3/3) | 85.0 (17/20) | 85.0 (17/20) | 100.0 (20/20) | 0 | 0 | 0 |
| Siemens | |  |  |  |  |  |  |  |  |  |  |
|  | Symphony | 1.5 T | 70.4 (19/27) | 85.2 (23/27) | 81.5 (22/27) | 0 | 0 | 0 | 66.4 (91/137) | 73.7 (101/137) | 77.4 (106/137) |
|  | Aera | 1.5 T | 50.0 (2/4) | 100.0 (4/4) | 75.0 (3/4) | 0 | 0 | 0 | 72.0 (18/25) | 52.0 (13/25) | 60.0 (15/25) |
|  | Skyra | 3.0 T | 100.0 (1/1) | 100.0 (1/1) | 100.0 (1/1) | 0 | 0 | 0 | 0 | 0 | 0 |
| CNN = convolutional neural network, MM = multidimensional. Data are percentages.  Data in parentheses are raw data (number of patients or aneurysms) used to calculate the percentages. | | | | | | | | | | | |

**Additional Experiments**

Additional experiments were conducted to investigate in detail the effect of the magnetic field strength of the MRI on the model performance in the external tests. In additional test 1, all models were trained with only 1.5-T data and tested with 3-T data. Similarly, all models were trained with only 3-T data and tested with 1.5-T data in additional test 2. For all models, additional test 1 with 3-T data resulted in higher sensitivity and lower false positives than additional test 2. This result suggests that differences in the magnetic field strength of MRA performed in the test can affect the experimental results.

**
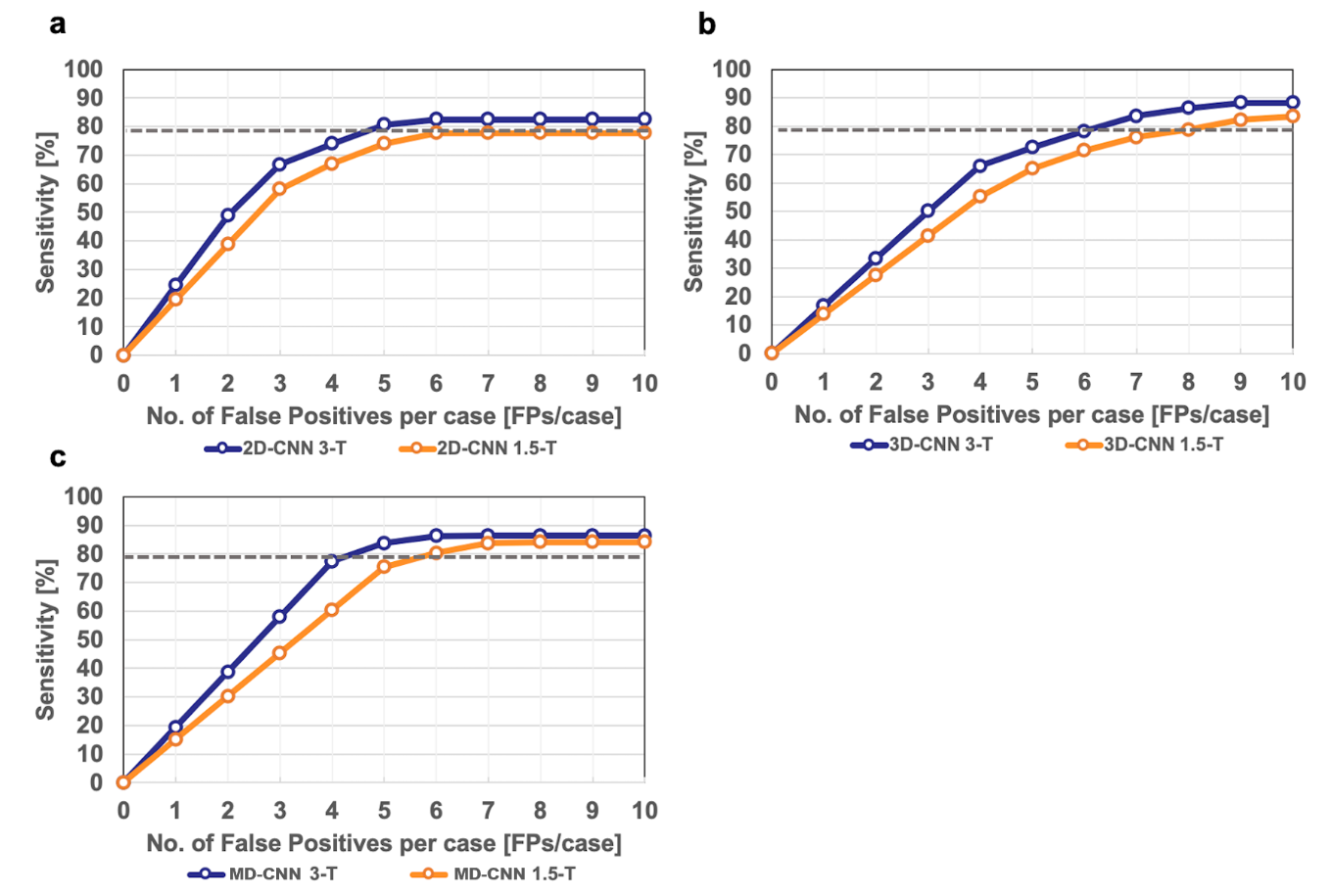
**

**Figure S2**: Free-response receiver operating characteristics of all models on the additional test 1 and 2. The blue line shows the results of additional test 1 and the orange line shows the results of additional test 2. The gray dashed line in each graph indicates a sensitivity of 80%.

CNN = convolutional neural network, MD = multidimensional.
